# Laparoscopic enterolithotomy for large gallstone ileus in an elderly high-risk patient: a case report

**DOI:** 10.1186/s12893-026-04045-1

**Published:** 2026-07-14

**Authors:** Marei Alshandeer

**Affiliations:** Department of General Surgery, King Khalid University, Abhā, Saudi Arabia

**Keywords:** Gallstone ileus, Mechanical bowel obstruction, Laparoscopic enterolithotomy, Geriatric surgery, Case report

## Abstract

**Background:**

Gallstone ileus is an uncommon complication of cholelithiasis leading to mechanical small bowel obstruction that typically affects geriatric populations with significant baseline medical conditions. The optimal management approach is highly debated, specifically when choosing between simple emergency enterolithotomy alone or a complex one-stage definitive biliary procedure in high-risk patients.

**Case presentation:**

A 79-year-old male with an ECOG performance status of 2, ASA Class IV risk classification, and known severe ischemic heart disease presented with acute mechanical small bowel obstruction. Preoperative contrast-enhanced computed tomography (CT) and subsequent emergency laparoscopic exploration identified an oversized, calcified intraluminal gallstone measuring 7 × 4 cm impacted within the terminal ileum. A laparoscopic enterolithotomy was successfully performed without a concomitant biliary intervention. The immediate postoperative course was complicated by an acute non-ST-elevation myocardial infarction (NSTEMI) detected via postoperative ECG. Following successful stabilization, the patient recovered gastrointestinal function and was safely discharged home after one week with structured outpatient cardiology follow-up.

**Conclusions:**

This case shows that an isolated laparoscopic enterolithotomy can serve as a technically feasible option for large gallstone ileus in selected high-risk patients when executed by experienced hands. However, clinicians must maintain extensive vigilance regarding perioperative cardiovascular strain, as the surgical resolution of obstruction must be balanced against severe potential cardiac complications.

## Background

Gallstone ileus represents a rare and clinically critical variant of mechanical small bowel obstruction, accounting for roughly 1–4% of all cases primarily within geriatric populations [1]. The disease develops as a late-stage manifestation of long-standing cholelithiasis, wherein an inflammatory adherence yields a biliary-enteric fistula—most commonly cholecystoduodenal—enabling large gallstones to migrate directly into the gastrointestinal lumen [2].

Radiologically, the condition is classically defined by Rigler’s triad, which encompasses the concurrent presentation of pneumobilia, mechanical bowel obstruction, and an ectopic intraluminal gallstone.

The condition is associated with high mortality and morbidity rates (ranging from 12% to 27%) due to advanced patient age, delayed clinical presentations, and substantial baseline co-morbidities [3, 4].

Management choices remain controversial. Traditional single-stage interventions historically combined enterolithotomy with concurrent cholecystectomy and fistula closure to address the primary obstruction and underlying biliary anatomy simultaneously. However, modern consensus supports a damage-control strategy in emergency settings [4, 5]. Attempting extensive biliary reconstruction during acute mechanical obstruction significantly increases operative times and induces prohibitive systemic stress, yielding high complication rates in highly comorbid populations.

We report a case of an emergency acute mechanical small bowel obstruction secondary to an oversized 7 × 4 cm gallstone in a 79-year-old cardiac patient. The case illustrates the balance between surgical relief of obstruction via isolated laparoscopic enterolithotomy and the management of a subsequent major postoperative cardiovascular complication.

## Case presentation

### Initial clinical profile and risk assessment

A 79-year-old male with a known history of severe ischemic heart disease and chronic symptomatic cholelithiasis presented to the emergency department with a 3-day history of progressive abdominal pain, bilious vomiting, marked abdominal distension, and complete obstipation. The patient had a baseline ECOG performance status of 2, remaining independent in activities of daily living but severely restricted during physical exertion. He was actively managed on maintenance beta-blockers (Metoprolol) and antiplatelets. There was no history of previous abdominal surgeries, and no previous CABG had been performed.

On clinical examination, the patient was in acute distress and uncomfortable but hemodynamically stable, with no preoperative signs of cardiogenic or hypovolemic shock. The abdomen was severely distended with generalized tenderness, marked tympany, and completely absent bowel sounds. There were no signs of generalized peritonitis, such as guarding or rebound tenderness. Physical examination also revealed baseline bilateral pitting pedal edema.

An urgent preoperative cardiac assessment was performed, revealing a baseline heart rate of 78 bpm and blood pressure of 128/74 mmHg. The preoperative ECG demonstrated baseline ischemic changes without acute ST-segment or T-wave abnormalities. Crucially, the preoperative baseline Troponin I level was documented at an exact value of 0.01 ng/mL, which was completely within normal physiological limits.

### Preoperative labs and diagnostic imaging

Admission laboratory testing was performed to establish an objective baseline profile (Table 1). Values demonstrated no significant leukocytosis, mild acute kidney injury secondary to dehydration, and a baseline serum lactate of 2.1 mmol/L, which was considered within normal limits and not elevated.

**Table 1:** Preoperative laboratory results

Laboratory parameter	Patient admission value	Reference range
White cell count (WBC)	9.8 × 10^9^ /L	4.0–11.0 × 10^9^ /L
Hemoglobin	13.4 g/dL	13.0–17.0 g/dL
Platelet count	185 × 10^9^ /L	150–450 × 10^9^ /L
Serum sodium	136 mEq/L	135–145 mEq/L
Serum potassium	4.1 mEq/L	3.5–5.1 mEq/L
Serum creatinine	1.4 mg/dL	0.7–1.2 mg/dL
Blood urea nitrogen (BUN)	28 mg/dL	0.7–1.2 mg/dL
Serum albumin	3.6 g/dL	3.5–5.0 g/dL
Total bilirubin	1.1 mg/dL	0.2–1.2 mg/dL
Serum lactate	2.1 mmol/L	0.5–2.2 mmol/L

**Fig. 1:**
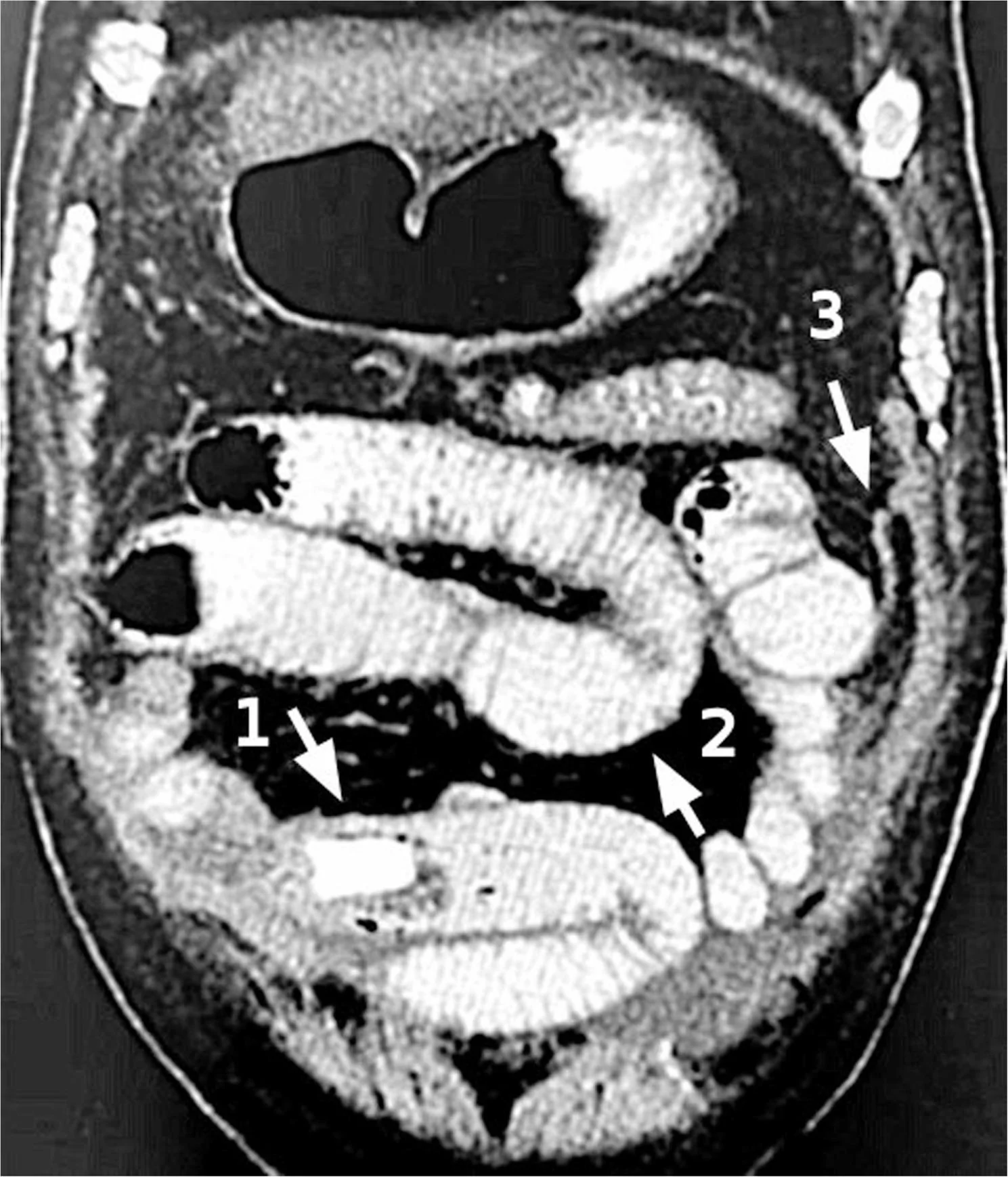
Coronal contrast-enhanced CT image demonstrating a distal mechanical small bowel obstruction with markedly dilated proximal small bowel loops and an oversized, calcified ectopic gallstone safely delineated within the distal ileum. White arrowheads denote the calcified gallstone [1], dilated small intestinal loops [2], and collapsed large bowel [3]

**Fig. 2:**
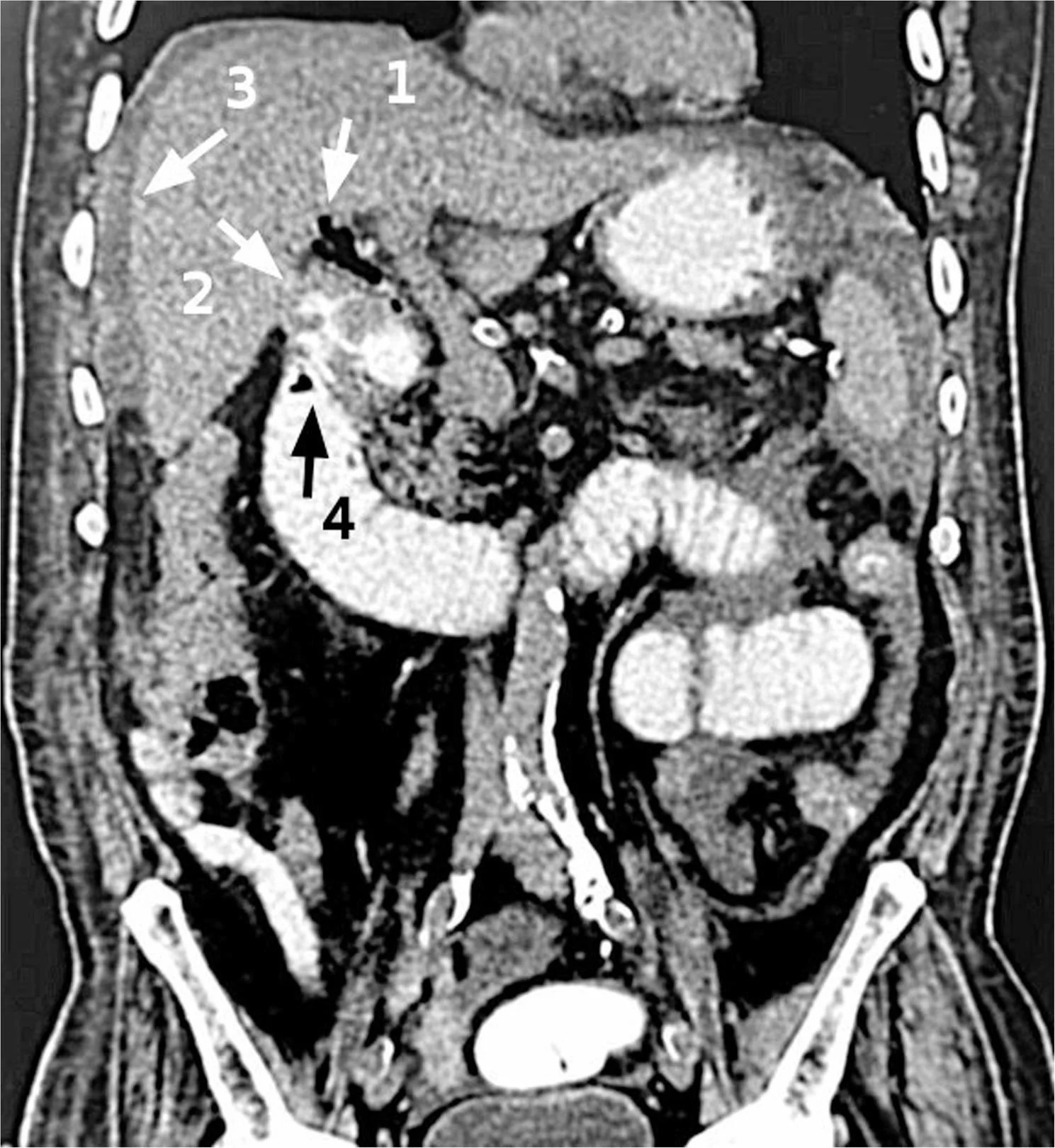
Additional coronal contrast-enhanced CT view demonstrating right upper quadrant pathology. White arrows denote central pneumobilia near the hepatic hilum [1] and the contracted, thick-walled gallbladder [2]. A third white arrow pinpoints a crescent of free perihepatic fluid representing ascites [3]. A contrasting black arrowhead highlights the suspected cholecystoduodenal fistula site [5] where the gallbladder directly abuts the adjacent duodenal lumen

**Fig. 3:**
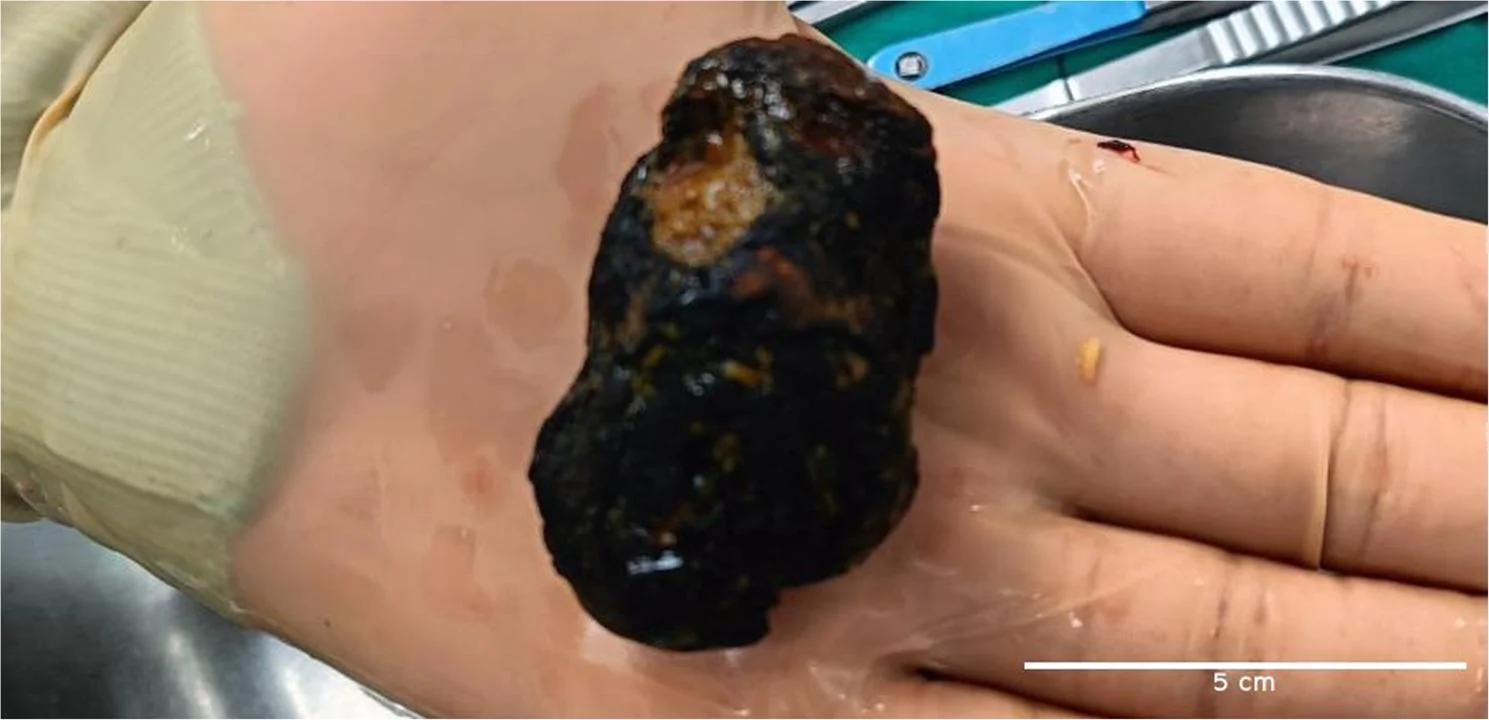
Extracted large gallstone ( 7x4 cm ) from the terminal ileum with a 5cm digital scale bar for comparison

Before obtaining advanced imaging, primary abdominal evaluation findings including clinical distension and typical signs of mechanical obstruction were noted on a baseline abdominal radiograph, which revealed multiple dilated small bowel loops with air-fluid levels. An urgent contrast-enhanced computed tomography (CT) scan of the abdomen and pelvis was then obtained directly to expedite diagnosis. The CT demonstrated definitive signs of Rigler’s triad: a large, calcified intraluminal calculus impacted within the terminal ileum, a severely contracted gallbladder with clear pneumobilia, and markedly dilated small bowel loops up to 4.5 cm with distal collapse of the large bowel loops (Figs. 1, 2 and 3). A cholecystoduodenal fistula tract was highly suspected based on these indirect signs.

The CT scan also revealed moderate free fluid/ascites within the pelvis, bilateral pleural effusions with basal atelectasis, and a mild pericardial effusion. Given the absence of peritonitis or free air, these fluid accumulations were determined to be systemic, driven by chronic volume overload and underlying ischemic cardiomyopathy rather than acute bowel necrosis or transmural perforation. A baseline preoperative echocardiogram confirmed severe left ventricular regional wall motion abnormalities with a depressed ejection fraction (EF) of 32%. The patient was classified as an ASA Class IV surgical candidate.

### Diagnostic challenges

No institutional or financial barriers to diagnostic access were encountered. The primary diagnostic challenge was clinical: distinguishing the patient’s systemic ascites and pleural effusions (caused by chronic heart failure) from acute localized fluid secondary to bowel necrosis. Rapid cross-sectional CT imaging successfully resolved this ambiguity without delaying surgery.

### Surgical intervention

An urgent operative intervention was planned. The patient underwent rapid perioperative cardiac optimization within the emergency unit, which involved central venous line placement for volume tracking, cautious titration of intravenous nitrates to mitigate afterload, and tight control of electrolytes to prevent malignant arrhythmias.

During pre-oxygenation and induction, the anesthetist was explicitly asked to perform active nasogastric tube (NGT) suction to decompress the stomach and proximal gastrointestinal tract, mitigating the high risk of pulmonary aspiration and reducing intra-abdominal volume.

Under general anesthesia, a laparoscopic approach was initiated via an open Hasson technique at the umbilicus to establish a carbon dioxide pneumoperitoneum at a restricted pressure of 12 mmHg. The patient was placed in the Trendelenburg position and tilted to the left. Port placement was meticulously arranged using a four-port design to optimize surgical logistics: A 10 mm umbilical port was established for housing a 30° laparoscope. A 5 mm suprapubic port was inserted to facilitate the use of the surgeon’s left hand. A 10 mm port in the Left Lower Quadrant (LLQ) was established to allow for the use of a 10 mm high-capacity suction instrument. A 5 mm port in the Left Upper Quadrant (LUQ) was placed to optimize assistance from the co-surgeon.

Laparoscopic inspection confirmed severely distended, edematous small bowel loops proximal to a hard mass lodged 30 cm proximal to the ileocecal valve. To prevent bowel wall injury or iatrogenic perforation during manipulation of the friable tissue, the bowel was retrogradely traced using atraumatic laparoscopic forceps. To isolate the field and achieve definitive bowel control, atraumatic clamps were placed both proximal and distal to the impacted stone.

Operating strictly within the peritoneal cavity, an endobag was introduced first through the 10 mm umbilical port and positioned near the field to be completely ready for immediate specimen acquisition. A small longitudinal enterotomy was then initiated on the antimesenteric border in the healthy tissue segment proximal to the impaction site. The instrument setup was quickly swapped out for a 10 mm high-volume suction tip to rapidly aspirate localized content and prevent any free intra-abdominal spillage. The enterotomy was then safely extended to match the dimensions of the calculus, allowing for the controlled extraction of the stone, which was immediately deposited into the waiting endobag to avoid any peritoneal contamination. The enterotomy was closed transversely in a standard two-layer fashion using absorbable 3 − 0 Vicryl sutures to preserve the luminal diameter. Complete patency was verified, and intraoperative assessment confirmed full bowel viability, marked by the return of a pink color and active peristalsis; no bowel resection was required. Explicit negative findings included the absolute absence of bowel perforation, transmural necrosis, or purulent peritonitis. A 19-Fr Jackson-Pratt drain was positioned in the right upper quadrant adjacent to the cholecystoduodenal fistula site. The stone-containing endobag was safely retrieved by extracting it through the 10-mm umbilical port site after extending the incision to the appropriate length.

Total operative time was 85 min with an estimated blood loss of 40 mL. No attempt was made to perform a concomitant biliary repair or cholecystectomy due to the severe baseline cardiac profile.

### Postoperative course and follow-up

Immediately following the procedure, the patient was shifted directly from the operating room to the Intensive Care Unit (ICU) for close hemodynamic monitoring. On the first postoperative day (POD 1), the patient experienced acute, substernal chest pain. A 12-lead postoperative ECG demonstrated dynamic 1.5-mm horizontal ST-segment depressions in leads V4–V6 and T-wave inversions, confirming an acute non-ST-elevation myocardial infarction (NSTEMI). Serial laboratory testing demonstrated a peak cardiac Troponin I level of 4.5 ng/mL.

Following the diagnosis of the NSTEMI, the patient was transferred to the Coronary Care Unit (CCU). Blood pressure was continuously monitored and maintained via a right radial arterial line to ensure target mean arterial pressures (MAP) above 65 mmHg. Following comprehensive cardiology consultation, invasive coronary angiography or intervention was deferred due to the immediate postoperative state and elevated bleeding risks. Instead, he was managed with a cautious, conservative pharmacological strategy consisting of dual antiplatelet therapy (Aspirin 81 mg daily and Clopidogrel 75 mg daily), therapeutic low-molecular-weight heparin (Enoxaparin 1 mg/kg every 12 h), and high-intensity statins. Systemic thrombolysis was explicitly avoided due to the prohibitive risk of immediate postoperative intra-abdominal hemorrhage. Transient hypotension was managed aggressively without vasopressors by optimizing cardiac preload under continuous telemetry.

Central venous pressure tracking guided the fluid management protocol to ensure a neutral fluid balance, preventing volume overload of the baseline failing ventricle while maintaining adequate renal perfusion. The patient was kept strictly nil per os (NPO) with a nasogastric tube on low intermittent suction until bowel function returned. The concurrent bilateral pleural effusions and mild basal atelectasis/consolidation observed on serial chest X-rays were managed conservatively with aggressive chest physiotherapy and targeted intravenous loop diuretics (Furosemide 20 mg intravenously twice daily), avoiding invasive thoracentesis.

The small, minimally invasive port incisions minimized abdominal wall trauma, which allowed the initiation of full antithrombotic and anticoagulation therapies on POD 1 with a minimized risk of a massive abdominal wound hematoma or fascial breakdown. Gastrointestinal function returned rapidly, with the patient passing flatus on POD 3, tolerating a clear liquid diet on POD 4, and achieving normal bowel function with the passage of stool on POD 5.

The patient stabilized cardiologically, was mobilized early out of bed by POD 4, and was safely discharged home on POD 7. This discharge was coordinated with the cardiology team, which arranged for an outpatient cardiology appointment, medication reconciliation, and a repeat echocardiogram.

Following discharge, the patient was closely monitored through regular clinical re-evaluations. He was evaluated in the outpatient general surgery clinic exactly one week following discharge and was subsequently seen again at the one-month postoperative mark. During these designated follow-up visits, the patient demonstrated complete clinical recovery, maintaining normal diet tolerance and bowel movements with zero reports of recurrent mechanical bowel obstruction, biliary colic, cholangitis, cholecystitis, or secondary cardiovascular events.

### Patient perspective

Due to the patient’s advanced age and the acute cardiac event on POD 1, a detailed retrospective interview was limited. However, during outpatient follow-up, the patient expressed satisfaction with the minimally invasive approach, noting that the minimal abdominal pain allowed him to tolerate early mobilization and adhere smoothly to his post-infarction medical regimen.

## Discussion

Gallstone ileus presents a high-stakes clinical dilemma, particularly in geriatric populations with extensive medical co-morbidities. This presentation is notable for an oversized 7 × 4 cm gallstone, which is significantly larger than the standard 2.0 to 2.5 cm threshold typically required to cause mechanical bowel obstruction. Stones of this magnitude are technically challenging to manipulate and carry a high risk of localized bowel wall erosion. The unique novelty of our case rests on the complex convergence of three clinical factors: the unusually large size of the impacted stone, the patient’s advanced age combined with severe baseline ischemic heart disease, and the successful navigation of a major postoperative myocardial infarction.

Our case adds to a growing body of minimally invasive literature by demonstrating that isolated laparoscopic enterolithotomy is a technically viable alternative to open surgery [6, 7]. However, the choice of a laparoscopic approach in an acute cardiac patient requires an understanding of periprocedural cardiovascular pathophysiology. The establishment of a carbon dioxide (CO_2_) pneumoperitoneum increases intra-abdominal pressure, which directly compresses the inferior vena cava, diminishes venous return, increases systemic vascular resistance, and reduces overall cardiac output. In patients with severe pre-existing ischemic cardiomyopathy, this increased afterload and diminished myocardial perfusion can precipitate a critical demand-ischemia mismatch (Type 2 myocardial infarction), which likely induced this patient’s postoperative myocardial infarction.

Despite triggering this cardiac event, the selection of a minimalist, laparoscopic approach provided the exact technical boundaries that enabled the patient to survive the complication. Had a conventional open laparotomy or a prolonged single-stage biliary reconstruction been pursued, the extended anesthesia time and extensive surgical trauma would have depleted the patient’s immediate physiological reserves, likely yielding a fatal outcome. Furthermore, contemporary database studies like NSQIP show that laparoscopy reduces surgical site infections and third-space fluid shifts compared to open alternatives [7]. The localized, small port-site wounds provided a stable abdominal wall that allowed the immediate and safe initiation of therapeutic anticoagulation and dual antiplatelet therapies on POD 1 with a minimized risk of a massive abdominal wound hematoma or fascial dehiscence. The small incisions also facilitated early out-of-bed mobilization, reducing hypostatic pneumonia risks.

Surgical choice heavily balances isolated enterolithotomy against a one-stage definitive procedure (comprising enterolithotomy, cholecystectomy, and fistula closure) [5, 8]. While a single-stage repair removes the underlying biliary tract pathology and prevents potential recurrent impaction, extensive literature indicates that leaving the fistula intact carries a long-term recurrence risk of less than 5% [8]. Many cholecystoenteric fistulas close spontaneously over time if the distal obstruction is fully cleared. Thus, an emergency single-stage approach should be restricted to low-risk, younger, hemodynamically stable candidates [4, 5]. In emergency scenarios involving high-risk geriatric patients, a strict “less is more” damage-control strategy must prevail, prioritizing rapid relief of the mechanical small bowel obstruction over technical completeness [5]. Treatment decisions must be based transparently on the patient’s clinical condition, the expected adverse consequences of not performing the surgery, and the risk of periprocedural adverse events. The patient and family were thoroughly counseled on the ~ 5% long-term recurrence risk, and any interval elective cholecystectomy was deferred due to baseline cardiac risk factors.

### Limitations

The primary limitation of this report is its single-patient format, which prevents the formulation of universal clinical guidelines or broader statistical safety profiles regarding laparoscopic parameters in cardiac populations. Additionally, the successful execution of an entirely intracorporeal enterolithotomy for an oversized stone requires advanced laparoscopic suturing skills and specialized institutional resources, which may not be universally available in every emergency center. Furthermore, we acknowledge the specific one-week and one-month follow-up visits as a chronological boundary of this short-term case report. However, this clinical tracking framework was sufficient and clear to document the successful resolution of all primary emergency parameters, including complete resolution of the mechanical small bowel obstruction, recovery of full gastrointestinal transit, and stabilization of the post-operative acute myocardial infarction.

## Conclusions

Laparoscopic enterolithotomy is a technically feasible management strategy for large gallstone ileus in selected high-risk geriatric patients when executed by an experienced team. However, this surgical approach must not be generalized as completely risk-free, as the systemic stress of CO_2_ pneumoperitoneum can induce severe postoperative cardiovascular complications in patients with low cardiac reserve. Surgical decision-making in these acute scenarios must be guided strictly by the patient’s immediate clinical status, the high mortality of an unmanaged mechanical bowel obstruction, and a realistic appraisal of periprocedural adverse risks.

## Data Availability

All data generated or analyzed during this case study are included within this published article.
